# Laparoscopic Splenectomy for Splenic Hamartoma: A Case Report

**DOI:** 10.1155/2012/435802

**Published:** 2012-10-22

**Authors:** Andrea Pisani Ceretti, Gabriele Bislenghi, Matteo Virdis, Nirvana Maroni, Andrea Gatti, Enrico Opocher

**Affiliations:** Department of General Surgery, San Paolo Hospital, University of Milan, Via Cesariano 10, 20154 Milano, Italy

## Abstract

Hamartoma is a rare splenic benign tumor usually accidentally detected as a radiologic finding. Preoperative diagnosis poses a challenge and thus surgery becomes necessary to confirm the clinical suspicion. Laparoscopic splenectomy has gained consensus as a standard surgical procedure particularly for autoimmune hematological diseases. This former experience has allowed this technique to be extended to other splenic pathologies. Here we report a case of total laparoscopic splenectomy for a bulky splenic hamartoma in a young male patient.

## 1. Introduction

Mass forming splenic tumors are rare. Malignancies include lymphoma and other less common lesions as primary splenic sarcoma, plasmacytoma, or metastatic disease from non-gastrointestinal tumor (e.g., lung melanoma or ovarian cancer) [1–7]. Benign lesions are exceedingly rare comprising mostly hemangiomas, cysts and inflammatory pseudotumors [8–12]. Splenic hamartoma is a very rare benign tumor with an incidence of 3 in 200000 splenectomies at a single center series [13] and 0,024% to 0,13% in an autopsies review [14]. Although hamartoma is benign and usually asymptomatic it is important to distinguish that from malignancy lesion. Medical imaging improvement has led an increase in the detection rate of incidentalomas of the spleen that pose a challenge, due to diagnostic difficulties [12]. As a consequence solitary splenic tumors may require splenectomy to assure a definitive histological diagnosis. Laparoscopic surgery has become the standard technique for both benign and malignant disease [15]. Minimally invasive surgery is particularly suitable for benign disease, enclosing hamartoma. Nevertheless to our knowledge only a few reports focusing on splenic hamartoma laparoscopic surgery have been published [16–19]. In this paper we describe a case of splenic hamartoma treated with totally laparoscopic approach.

## 2. Case Report

A 28-year-old Italian male presented at the ER in August 2011 with diarrhea and an antibiotics refractory fever. Laboratory findings did not show any significant problem, apart from a mild hypertransaminasemia: (ALT 65 u/L AST 46 u/L).

The patient was discharged with the indication for further investigations. Abdominal ultrasonography was performed two weeks later, showing normal liver, biliary ducts, and pancreas morphology. Nevertheless a 15 cm increased spleen was found, containing in its cephalic portion a hypoechoic and asymptomatic 70 × 77 mm solid mass. Color Doppler showed blood flow along the edge of the round mass, without relevant signals within the neoformation.

**Figure 1:**
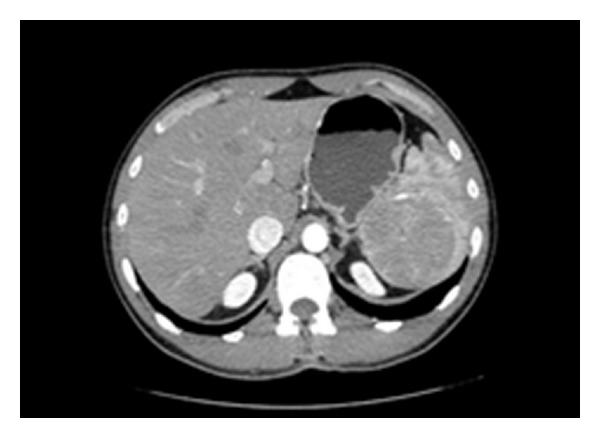
Computed tomography showing a hypodense mass of the spleen with contrast enhancement during arterial phase.

CT scan was also executed, to better evaluate the splenic lesion: images before contrast material administration displayed a round mass, hypodense compared to the normal splenic parenchyma. After injection of intravenous contrast, it showed a mild contrast enhancement during the arterial phase, followed by a complete washout during the late venous phase, without relevant vascular components inside (Figure 1).

After these preoperative findings suggestive but not definitive for a well-defined histological lesion, considering the high risk for splenic bleeding in the case of biopsy and the risk for spontaneous rupture, the patient was candidate for totally laparoscopic splenectomy two months after first clinical evaluation. Routine preoperative examination, chest X-ray, blood test, and anaesthesiologic evaluation did not set any contraindication to the operation.

The patient was placed in a 30-degree right lateral decubitus, with the help of a cushion positioned along his back.

**Figure 2:**
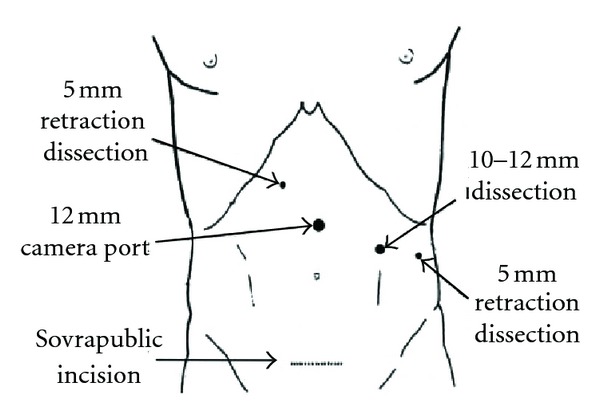
Disposition of trocars for laparoscopic splenectomy.

Right arm was arranged for intraoperative intravenous fluid infusion, whereas left arm was suspended with bendages above patient's head to allow full access to the left hemiabdomen. A 12 mm disposable Hasson's trocar was placed using an open technique in supraumbilical region for the first induction of pneumoperitoneum and for optical access. A second 10–12 mm disposable trocar was positioned in left hypochondrium, at the intersection between midclavicular line and infracostal line and two more 5 mm accesses, the first in left flank, along anterior axillary line and the second in right hypochondrium, along midclavicular line (Figure 2). Laparoscopic exploration of the abdominal cavity was negative for any associated or generalized pathology, as well as for accessory spleen. An enlarged 15 cm spleen was identified; omental adhesions and splenocolic ligament were dissected using monopolar and ultrasonic dissector in order to lower the splenic flexure of the colon and to expose the inferior pole of the organ. Dissection was carried on with the section of the inferior and superior gastrosplenic vessels. Thus the lesser peritoneal sac was open, exposing the anterior side of the splenic hilum and the pancreas tail; the stomach was retracted toward the patient's rightside. Vascular splenic pedicle was then isolated at the hilum allowing identification of periferic branches of splenic artery and vein.

Vessels were finally dissected using two vascular 30 mm endostapler refills (EndoGIA, Autosuture, Covidien, Mansfield, MA, USA) obtaining a perfect hemostasis. Spleen was then mobilized toward the patient's right side, to allow dissection of the posterior peritoneal reflection or splenorenal ligament. After placing the totally freed organ inside an EndoBag (EndoCatch II, Autosuture, Covidien, Mansfield, MA, USA) a Pfannestiel suprapubic incision was performed to remove the specimen. The peritoneal-sac access was carried on with a partial surgical section of the rectus muscle in order to allow an easier removal of the unbroken specimen. After clearing the spleen, laparotomic incision was sealed in multiple layers. An accurate hemostasis control and a generous abdominal cavity washing were performed after the recreation of pneumoperitoneum; a single drainage was positioned in the left subphrenic space and major accesses were finally closed.

A short-term perioperative antibiotic prophylaxis based on cefazolin (1 gr every 8 hours) was administered for the first 24 hours. Pain control was always optimal during the entire hospitalization: during the first 24 hours after surgery pain control was managed with opiates and paracetamol; on the following day opiates were replaced with anti-inflammatory nonsteroidal drugs removed from the 3rd day on; during the same day the drainage was removed. The patient was allowed to eat from the 2nd postoperative day. The complete functionality of the gastrointestinal tract was regained between the 3rd and 4th postoperative day. The patient was dismissed on the 5th day after surgery and stitches were removed 6 days later during an outpatient clinical control. Vaccination against Streptococcus pneumoniae, Haemophilus influenza type B, and Neisseria meningitidis infections was administered 20 days after surgery. Perioperative anticoagulant prophylaxis with subcutaneous heparin (4000 I.U. per day) was applied and continued for 4 weeks after surgery.

**Figure 3:**
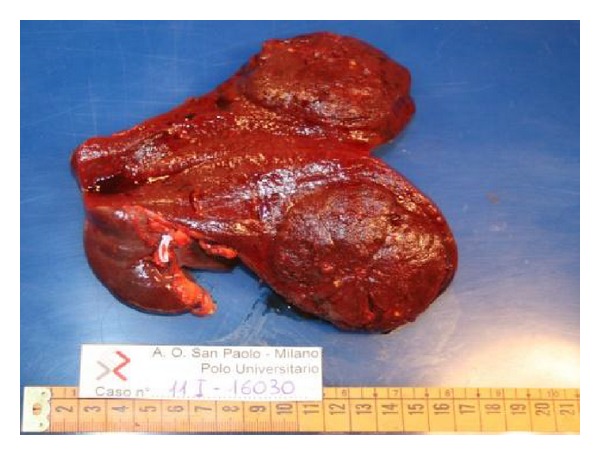
The removed specimen: spleen with a round capsulated nodule of increased stiffness.

The anatomopathological analysis of the spleen showed a 14 × 7 × 8 cm organ, weighting 430 gr, containing a 7,5 cm reddish brown nodule of slightly augmented stiffness, compared to the surrounding splenic tissue (Figure 3). The histopathological diagnosis confirmed the original suspect of splenic hamartoma, also supported by the immunohistochemical stains positive for CD-8 and negative for CD-34.

## 3. Discussion

### 3.1. Differential Diagnosis

Solid lesions of the spleen are quite rare and generally asymptomatic. About half of these are accidentally detected during imaging studies carried out for unrelated causes [18]. Differential diagnosis includes primary malignancies, in particular non-Hodgkin's lymphoma and angiosarcoma, which is the most common nonlymphoid malignant primary tumor of the spleen [20]. Lung cancer, melanoma, ovary, uterine cervix, and other nongastrointestinal tumors splenic metastasis are also reported [21, 22]. Benign lesions are extremely rare (7/100000 autopsies) and have generally vascular origin [8]. Hemangioma, littoral cell angioma, lymphangioma, hemangioendothelioma, and hamartoma of the spleen have been previously described in the literature [2–8]. Inflammatory miofibroblastic tumor, disseminated fungal or mycobacterial infections, and sarcoidosis are also included in the differential diagnosis [23].

Splenic hamartoma is a rare benign tumor which occurs in any age group and has the same male and female occurrence. Its size tends to be longer in females, probably due to hormonal influence on tumor growth [24–26]. The tumor is usually detected as a singular lesion with a diameter ranging from a few millimeters to several centimeters. Symptoms such as pain, palpable mass, or spontaneous rupture are associated with longer lesions. Hypersplenism, including thrombocytopenia, anemia, pancytopenia, or malignant hematologic conditions, is described even if uncommon [27–29].

### 3.2. Imaging

It is difficult to make a definitive preoperative diagnosis basing on conventional imaging including ultrasound and CT scan. Generally splenic hamartomas present as homogeneous, hypoechoic masses with multiple radial blood-flow signals at Color Doppler sonograms [30]. Contrast-enhanced sonography with microbubble contrast agents shows that tumor could be markedly enhanced [31]. CT scan displays a hypoechoic lesion with a diffuse heterogeneous enhancement after intravenous administration of contrast material [32].

### 3.3. Fine Needle Aspiration Biopsy

Fine needle aspiration biopsy may be useful to establish a pathological diagnosis. However this technique is associated with some dreadful complications including bleeding and abdominal seeding. These are the reasons why this procedure has been rarely reported [24, 33, 34].

### 3.4. Histology

Histologically hamartoma presents as an overgrowth of the normal spleen components [33]. Microscopically there are two types of splenic hamartoma: follicular and pulposal types. Splenic hamartoma is either of the pulposal type resembling a splenic red pulp or a lymphoid type resembling a white pulp. Symptomatic cases are invariably of the red pulposal variety because of an increased number of vascular channels which can sequester blood cells and produce hypersplenism [13]. Clinical diagnosis may be confirmed by immunopathological examination. Immunohistochemically endothelial cells of hamartoma are CD8-positive and CD34-negative in contrast to the CD8 negative and CD34 positive endothelial cells of hemangioma [34].

### 3.5. Surgery

Laparoscopic splenectomy (LS) is the standard procedure for most of benign and malignant hematologic diseases [15]. The laparoscopic approach reduces postoperative complications, in particular pulmonary ones such as pneumonia and atelectasis as well as intra-abdominal and wound infections and shortens recovery [35]. Nevertheless massive splenomegaly, defined as maximum diameter exceeding 20 cm, represents a contraindication for laparoscopy because in this case surgery may be more difficult, thus requiring significant experience [35]. Complications following LS generally relate with the size of the spleen regardless of the underlying disease. A patient selection based on splenic size on preoperative imaging is thus necessary. Moreover LS has become the procedure of choice not only for the management of hematologic diseases [36] but also for the management of solid splenic tumors [11]. In these cases splenectomy is performed in terms of diagnostic and staging procedure considering the impossibility to make a definitive preoperative diagnosis. Thus removal of the intact organ for pathologic examination becomes necessary, being a further difficulty in laparoscopic approach [37, 38]. On this issue partial splenectomy is reported in the literature as a possible alternative surgical procedure for benign cysts and in the setting of unclear splenomegaly or inconclusive blood and bone-marrow findings. It is mainly performed in children to preserve the splenic immunologic function and its important role in immune defense [39, 40]. Even splenic benign tumors, including vascular neoplasms, may be suitable for partial splenectomy. Nevertheless the impossibility in most of these cases to assure a definitive preoperative diagnosis in addition to technical limits, a higher risk of complications (rupture, haemorrhage) and recurrences does not allow this surgery to be routinely and entensively used. There are no large series on LS for solid splenic lesions [18]. This is probably justified by the rarity of this entity and by a diffuse reluctance in treating them laparoscopically, specifically when they are associated with splenomegaly. In particular only a few cases of laparoscopic procedure for splenic hamartoma have been reported in detail. In all these cases a hand-assisted laparoscopic splenectomy (HALS) was performed [16, 17, 19]. The use of HALS technique offers the undeniable advantage of the palpation and detection of malignant lesions involving adjacent structures (regional lymph nodes, pancreas, and stomach). It also facilitates the identification of correct planes and mobilization of the spleen by using the hand as a retractor. In the case of bleeding it can be easily controlled by manual compression of the splenic vascular pedicle [37, 41–43]. Despite these undeniable benefits, the constant improvement of technique in mini-invasive surgery and the remarkable innovation of instruments have allowed the diffusion of total laparoscopic approach as a safe procedure for splenectomy. Thereby most of the cases of splenic tumors, particularly those with the lack of malignancy at preoperative clinical and radiological examination, are suitable for a total laparoscopic resection and HALS may be consequently reserved only to those tumors with associated splenomegaly. As a result of these considerations, thanks to the experience gained in LS for hematologic disorders (e.g., autoimmune thrombocytopenia, autoimmune hemolytic anemia), in the present case we performed a total LS for splenic hamartoma. A satisfactory result was achieved in terms of operative time, blood loss, pain control, early hospital discharge, and cosmetic results.

## 4. Conclusion

In conclusion the rapid advancement of imaging modalities leads to the identification of more asymptomatic lesions of the spleen. In most of these cases malignancy cannot be ruled out by preoperative clinical and radiological evaluation and surgery becomes necessary. In the absence of massive splenomegaly total laparoscopic splenectomy may be a safe and feasible procedure for the diagnosis and treatment of both benign and malignant solid tumors of the spleen as demonstrated by this particular case of splenic hamartoma.

## References

[1] Bostick W (1945). Primary splenic neoplasm. *The American Journal of Pathology*.

[2] Walsh RM, Heniford BT (1999). Laparoscopic splenectomy for non-Hodgkin lymphoma. *Journal of Surgical Oncology*.

[3] Otrock ZK, Seoud MA, Khalifeh MJ, Makarem JA, Shamseddine AI (2006). Laparoscopic splenectomy for isolated parenchymal splenic metastasis of ovarian cancer. *International Journal of Gynecological Cancer*.

[4] Pang LC (2004). Solitary recurrent metastasis of squamous cell carcinoma of the uterine cervix in the spleen: case report. *Southern Medical Journal*.

[5] Raval MV, Zemon H, Kumar SS, Brody FJ (2005). Laparoscopic splenectomy for metastatic squamous cell cancer of the neck. *Journal of Laparoendoscopic and Advanced Surgical Techniques*.

[6] Takahashi H, Yano H, Monden T, Kinoshita T (2004). Hand-assisted laparoscopic splenectomy for solitary splenic metastasis from uterine corpus carcinoma. *Surgical Endoscopy*.

[7] Yano H, Iwazawa T, Kinuta M (2002). Solitary splenic metastasis from ovarian cancer successfully treated by hand-assisted laparoscopic splenectomy: report of a case. *Surgery Today*.

[8] Moriyama S, Inayoshi A, Kurano R (2000). Inflammatory pseudotumor of the spleen: report of a case. *Surgery Today*.

[9] Blansfield JA, Goldhahn RT, Josloff RK (2005). Littoral cell angioma of the spleen treated by laparoscopic splenectomy. *Journal of the Society of Laparoendoscopic Surgeons*.

[10] Kwon AH, Inui H, Tsuji K, Takai S, Imamura A, Kamiyama Y (2001). Laparoscopic splenectomy for a lymphangioma of the spleen: report of a case. *Surgery Today*.

[11] Yano H, Imasato M, Monden T, Okamoto S (2003). Hand-assisted laparoscopic splenectomy for splenic vascular tumors: report of two cases. *Surgical Laparoscopy, Endoscopy and Percutaneous Techniques*.

[12] Shapiro AJ, Adams ED (2006). Inflammatory pseudotumor of the spleen managed laparoscopically. Can preoperative imaging establish the diagnosis? Case report and literature review. *Surgical Laparoscopy, Endoscopy and Percutaneous Techniques*.

[13] Silverman ML, LiVolsi VA (1978). Splenic hamartoma. *American Journal of Clinical Pathology*.

[14] Lam KY, Yip KH, Peh WCG (1999). Splenic vascular lesions: unusual features and a review of the literature. *Australian and New Zealand Journal of Surgery*.

[15] Habermalz B, Sauerland S, Decker G (2008). Laparoscopic splenectomy: the clinical practice guidelines of the European Association for Endoscopic Surgery (EAES). *Surgical Endoscopy and Other Interventional Techniques*.

[16] Namikawa T, Kitagawa H, Iwabu J, Kobayashi M, Matsumoto M (2010). Laparoscopic splenectomy for splenic hamartoma: case management and clinical consequences. *World Journal of Gastrointestinal Surgery*.

[17] Tatekawa Y, Kanehiro H, Nakajima Y (2007). Laparoscopic extirpation of splenic hamartoma. *Pediatric surgery international*.

[18] Makrin V, Avital S, White I, Sagie B, Szold A (2008). Laparoscopic splenectomy for solitary splenic tumors. *Surgical Endoscopy and Other Interventional Techniques*.

[19] Yoshizumi T, Iso Y, Yasunaga C, Kitano S, Sugimachi K (1997). Laparoscopic splenectomy for splenic hamartoma. *Surgical Endoscopy*.

[20] Walsh RM, Heniford BT (1999). Laparoscopic splenectomy for non-Hodgkin lymphoma. *Journal of Surgical Oncology*.

[21] Berge T (1974). Splenic metastases: frequencies and patterns. *Acta Pathologica et Microbiologica Scandinavica*.

[22] Morgenstern L, Rosenberg J, Geller SA (1985). Tumors of the spleen. *World Journal of Surgery*.

[23] Cosme Á, Tejada Á, Bujanda L (2007). Littoral-cell angioma of the spleen: a case report. *World Journal of Gastroenterology*.

[24] Lee H, Maeda K (2009). Hamartoma of the spleen. *Archives of Pathology and Laboratory Medicine*.

[25] Jia HB, Li YP, Han DE (2006). Splenic hamartoma: case report and review of literature. *Chinese Medical Journal*.

[26] Yu RS, Zhang SZ, Hua JM (2004). Imaging findings of splenic hamartoma. *World Journal of Gastroenterology*.

[27] Abramowsky C, Alvarado C, Bradley Wyly J, Ricketts R (2004). Hamartoma" of the spleen (splenoma) in children. *Pediatric and Developmental Pathology*.

[28] Hayes TC, Britton HA, Mewborne EB, Troyer DA, Saldivar VA, Ratner IA (1998). Symptomatic splenic hamartoma: case report and literature review. *Pediatrics*.

[29] Wirbel RJ, Uhlig U, Futterer KM (1996). Case report: splenic hamartoma with hematologic disorders. *American Journal of the Medical Sciences*.

[30] Tang S, Shimizu T, Kikuchi Y, Shinya S, Kishimoto R, Fujioka Y (2000). Color Doppler sonographic findings in splenic hamartoma. *Journal of Clinical Ultrasound*.

[31] Nakanishi S, Shiraki K, Yamamoto K, Nakano T, Koyama M, Yano T (1991). Basket pattern blood flow signals discovered in a case of splenic hemangioma: value of compressing the tumor. *American Journal of Roentgenology*.

[32] Ohtomo K, Fukuda H, Mori K, Minami M, Itai Y, Inoue Y (1992). CT and MR appearances of splenic hamartoma. *Journal of Computer Assisted Tomography*.

[33] Falk S, Stutte HJ (1989). Hamartomas of the spleen: a study of 20 biopsy cases. *Histopathology*.

[34] Zukerberg LR, Kaynor BL, Silverman ML, Harris NL (1991). Splenic hamartoma and capillary hemangioma are distinct entities: immunohistochemical analysis of CD8 expression by endothelial cells. *Human Pathology*.

[35] Casaccia M, Torelli P, Squarcia S (2006). Laparoscopic splenectomy for hematologic diseases: a preliminary analysis performed on the Italian Registry of Laparoscopic Surgery of the Spleen (IRLSS). *Surgical Endoscopy and Other Interventional Techniques*.

[36] Knauer EM, Ailawadi G, Yahanda A (2003). 101 Laparoscopic splenectomies for the treatment of benign and malignant hematologic disorders. *American Journal of Surgery*.

[37] Heniford BT, Matthews BD, Answini GA, Walsh RM (2000). Laparoscopic splenectomy for malignant diseases. *Seminars in Laparoscopic Surgery*.

[38] Schlachta CM, Poulin EC, Mamazza J (1999). Laparoscopic splenectomy for hematologic malignancies. *Surgical Endoscopy*.

[39] Uranues S, Grossman D, Ludwig L, Bergamaschi R (2007). Laparoscopic partial splenectomy. *Surgical Endoscopy and Other Interventional Techniques*.

[40] Héry G, Becmeur F, Méfat L (2008). Laparoscopic Partial Splenectomy: indications and results of a multicenter retrospective study. *Surgical Endoscopy and Other Interventional Techniques*.

[41] Walsh RM, Heniford BT, Brody F, Ponsky J (2001). The ascendance of laparoscopic splenectomy. *American Surgeon*.

[42] Heniford BT, Park A, Walsh RM (2001). Laparoscopic splenectomy in patients with normal-sized spleens versus splenomegaly: does size matter?. *American Surgeon*.

[43] Kercher KW, Matthews BD, Walsh RM, Sing RF, Backus CL, Heniford BT (2002). Laparoscopic splenectomy for massive splenomegaly. *American Journal of Surgery*.

